# Feasibility of Physical Exam and Performance‐Based Tests in Individuals With Chronic Low Back Pain: A Descriptive Study

**DOI:** 10.1002/jsp2.70096

**Published:** 2025-08-12

**Authors:** Sara R. Piva, Zakiy Alfikri, William Anderst, Kevin M. Bell, Cristiane Carlesso, Jessa Darwin, Anthony Delitto, Carol M. Greco, Marit E. Johnson, Gina P. McKernan, Rachel McLoughlin, Charity G. Patterson, Rachel E. Roos, Michael J. Schneider, Clair Smith, Gwendolyn A. Sowa, Nam V. Vo, Leming Zhou

**Affiliations:** ^1^ Department of Physical Therapy University of Pittsburgh School of Health and Rehabilitation Science Pittsburgh Pennsylvania USA; ^2^ Clinical and Translational Science Institute University of Pittsburgh Pittsburgh Pennsylvania USA; ^3^ Department of Bioengineering University of Pittsburgh Swanson School of Engineering Pittsburgh Pennsylvania USA; ^4^ Department of Orthopaedic Surgery University of Pittsburgh School of Medicine Pittsburgh Pennsylvania USA; ^5^ Department of Physical Medicine and Rehabilitation University of Pittsburgh School of Medicine Pittsburgh Pennsylvania USA; ^6^ Department of Psychiatry University of Pittsburgh School of Medicine Pittsburgh Pennsylvania USA; ^7^ Department of Biomedical Informatics University of Pittsburgh School of Medicine Pittsburgh Pennsylvania USA; ^8^ Doctor of Chiropractic Program University of Pittsburgh School of Health and Rehabilitation Science Pittsburgh Pennsylvania USA; ^9^ Ferguson Laboratory for Orthopaedic and Spine Research Department of Orthopaedic Surgery, University of Pittsburgh School of Medicine Pittsburgh Pennsylvania USA; ^10^ Department of Pathology University of Pittsburgh School of Medicine Pittsburgh Pennsylvania USA; ^11^ McGowan Institute University of Pittsburgh Pittsburgh Pennsylvania USA; ^12^ Department of Health Information Management University of Pittsburgh School of Health and Rehabilitation Sciences Pittsburgh Pennsylvania USA; ^13^ Intelligent Systems Program University of Pittsburgh School of Computing and Information Pittsburgh Pennsylvania USA

**Keywords:** chronic low back pain, feasibility, muscle function, performance‐based tests, physical examination, physical function, special tests

## Abstract

**Background:**

Despite the wide utilization of physical tests and pain assessments to evaluate individuals with chronic low back pain (cLBP), there is limited information about their feasibility in terms of test duration, the ability of individuals with cLBP to perform these tests, and associated adverse events. The literature also lacks reports on comprehensive characterization of physical tests to serve as a reference for clinicians and researchers. The objectives of the present work are to assess the feasibility of a comprehensive battery of physical tests and pain assessments germane to individuals with cLBP and characterize the tests' values in the context of a large cohort.

**Methods:**

This cross‐sectional analysis uses enrollment data from a large observational study conducted by the University of Pittsburgh Mechanistic Research Center—“Low Back Pain: Biological, Biomechanical, Behavioral Phenotypes (LB^3^P).” LB^3^P is part of the National Institutes of Health's Helping to End Addiction Long‐term Initiative. Individuals with cLBP were screened by trained clinicians who assessed their safety to partake in up to 37 physical tests based on pre‐existing medical conditions. Testers could elect not to administer tests based on their clinical judgment and participants could refuse to partake in tests. The reasons for not performing tests were recorded. The feasibility of the tests was assessed by the time to complete each test, percentages and reasons for tests not done, and adverse events related to test performance. Descriptive statistics for the physical tests were computed for the sample overall, and for the subgroups (male/female and age < 60/≥ 60) to serve as reference values for individuals with cLBP.

**Results:**

The testing protocol took on average 130 min. In total, 8.9% of tests were not done. About one third of tests not done were screened out due to medical conditions identified during the safety screening, and two‐thirds due to the tester's clinical judgment or participant refusal. Only four adverse events occurred, and they resolved without sequelae. The tests most often omitted were those requiring maximal and submaximal physical effort or could elevate blood pressure in those with hypertension, such as muscle strength testing of the hip, abdomen, and thigh, or hand immersion in cold water. From the 1007 participants enrolled in the study, those who did not complete one or more tests tended to be older, obese, less educated, and experienced more disability and back pain for a longer time. The descriptive statistics of the 37 tests are reported stratified by sex and age.

**Conclusions:**

The results support the safety and feasibility of a comprehensive battery of physical tests and pain assessments in individuals with cLBP. This study also provides novel information on the test's performance frequency, reasons for not being completed, duration, and descriptive results in individuals with cLBP. This comprehensive characterization provides reference values for comparison in future research planning and clinical practice.

## Graphical abstract

As a part of the University of Pittsburgh LB3P Mechanistic Research Center, this study used ecological momentary assessment (EMA) and wearable devices to collect real‐time data in a large cohort of adults with cLBP. The primary aims were to collect and characterize pain and activity profiles of individuals with cLBP.

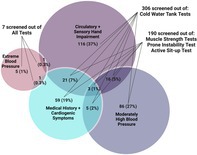

## 1 Introduction

Chronic low back pain (cLBP) is a leading cause of disability globally with far‐reaching socioeconomic consequences [1, 2]. Considering the multidimensionality of cLBP, attempting to phenotype these patients requires collecting large amounts of data, which may impose substantial participant burden. The burden for collecting phenotypic data is specific to the type of data. For example, collecting biological samples such as blood, urine, saliva, and stool, while a nuisance for some participants, takes a few minutes and tends to be well tolerated. Collecting data on behavioral factors mainly involves completing surveys and is generally acceptable to participants. On the other hand, obtaining data from an extensive battery of physical tests and assessing movement‐evoked pain not only takes considerable time, but also may exacerbate back pain due to the taxing movement repetition and physical effort required to complete these tests.

Despite the wide utilization of physical tests and pain assessments in daily practice and research, there is limited information about their feasibility regarding test duration, ease of performance, and associated adverse events in individuals with cLBP. The existing literature also fails to provide a comprehensive characterization of physical tests to serve as a reference for clinicians and researchers. To that end, the objectives of the present work are to assess the feasibility of a comprehensive battery of physical tests and pain assessments germane to individuals with cLBP, and to characterize their value in the context of a large cohort. Considering that sex may play a role in test performance and older individuals may present more severe physical limitations performing these tests [3, 4, 5], we stratified the results based on sex at birth and age.

## 2 Materials and Methods

This descriptive study reports cross‐sectional data from the University of Pittsburgh's Low Back Pain: Biological, Biomechanical, Behavioral Phenotypes (LB^3^P) Mechanistic Research Center. The LB^3^P is a member of the National Institutes of Health's (NIH) Back Pain Consortium (BACPAC) Research Program—which is part of the Helping to End Addiction Long‐term (HEAL) Initiative (1U19AR076725) [6]. The LB^3^P study enrolled 1007 adults with cLBP who were identified during their routine clinical care or through research registries and public announcements. Enrollment occurred from November 2020 to March 2024, and all participants completed informed consent procedures as approved by the University of Pittsburgh Institutional Review Board. The study was monitored by an independent observational study monitoring board and an advisory panel. This report follows the STrengthening the Reporting of OBservational studies in Epidemiology (STROBE) reporting guidelines.

### 2.1 Participants

Participants were required to be English‐speaking adults who reported having cLBP, defined as pain located between the inferior border of the ribcage and gluteal fold lasting for greater than 3 months and experienced for at least half the days in 6 months prior to screening [7]. Individuals were excluded if they were not identified in the University of Pittsburgh Medical Center Electronic Health Record system, were participating in a masked intervention study for LBP, or had a medical condition that would place them at risk or preclude them from complying with study procedures.

### 2.2 Measures

The test battery described in this work was performed as one of the components of the enrollment visit for the LB^3^P study. The entire visit was approximately 4.5 h long and involved consenting, collecting biological samples, completing surveys, and the battery of physical tests and pain assessments. Participants were compensated for their time. The tests were administered by protocol‐certified research physical therapists (PTs) from the Department of Physical Therapy—Clinical and Translational Research Center, a clinical center part of the University of Pittsburgh Clinical and Translational Science Institute. The clinic was kept at a constant temperature of about 23°C with minimal noise. Each tester received approximately 16 h of training to standardize testing procedures and data collection methods. Fidelity checks of testing procedures entailed in‐person observation and took place every 6 months throughout the study duration.

The physical tests were selected by multidisciplinary professionals such as PTs, chiropractors, physical medicine and rehabilitation physicians, bioengineers, and pain experts. The rationale for selecting these tests included their relative simplicity, not requiring expensive equipment, and being commonly used in research or clinical practice. These tests were standardized and harmonized with the other research studies funded by the BACPAC Research Program. To minimize fatigue, the tests were administered in a consistent order in a manner that minimized changes in participants' positions. For example, several seated tests were performed back‐to‐back, followed by standing tests, and then tests that required lying down. The participants were also provided with water, snacks, and periods of rest as needed. Although the aim was to complete all testing within a single visit, provisions were proactively made to split the visit into two if needed. The detailed description of all tests along with their sequence of performance, scoring, and interpretation is provided in Table 1.

**TABLE 1:** Description of physical tests and their order of administration.

Test name (order of administration)	Test description	Test scoring/Recording
Neurological examination		
Sensation^a^ [8] (1)	Participant sitting, tester applies light touch over anteromedial lower leg (L4), dorsum of foot (L5), and posterolateral lower leg and foot (S1).	Normal sensation Abnormal (no/diminished sensation or hypersensitive)
Deep Tendon Reflex^a^ [8] (2)	Participant sitting, tester uses a reflex hammer to tap on the patellar tendon, Achilles tendon, and medial hamstring tendon.	Normal reflex Abnormal (absent/diminished or hyperactive reflex)
Babinski Reflex^a^ [9] (3)	Participant sitting, tester uses the dull point of a reflex hammer to firmly stroke along the lateral plantar side of the foot from heel to toe and across the metatarsal pads to the base of the big toe.	Normal—Downgoing (all toes flex) Abnormal—Upgoing (great toe extends, and lateral toes fan out)
Myotome Testing (traditional score)^a^ [8] (4)	Participant sitting, tester performs manual muscle testing of: hip flexion (L2/L3) knee extension (L3/L4) ankle dorsiflexion (L4) great toe extensor (L5) knee flexion (S2)	Abnormal‐ no contraction OR trace of contraction with minimal/no joint motion OR active movement with gravity eliminated OR active movement against gravity, but not resistance, OR active movement against gravity with some resistance Normal– active movement against gravity with full resistance.
Myotome Testing (repetition score)^a^ [10] (7)	Single‐Leg Calf Raise (S1)—Participant standing on the testing leg, lifts heel as high as possible and lowers back down.	Abnormal—No contraction OR muscle contraction but no movement OR partial ROM, less than 1 quality repetition OR full ROM and 1 quality repetition OR full ROM and 2–3 quality repetitions Normal—Full ROM and 4–5 quality repetitions
Slump Test^a^ [11, 12] (5)	Participant sitting with spine erect, hands behind back, and knees bent to 90° over the table edge. Participant slumps trunk with chin tucked in, extends knee, and dorsiflexes ankle. 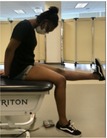	Positive neural tension: radicular symptoms in the buttock/lower extremity are felt with increased neural tension and decreased with less tension
Passive Straight Leg Raise and Crossed Straight Leg Raise^a^ [11, 13] (8) Tightness of Posterior Lower Extremity^a^ [14] Muscles [15]	Participant in supine, tester passively dorsiflexes ankle and lifts leg off the table while keeping the knee extended. 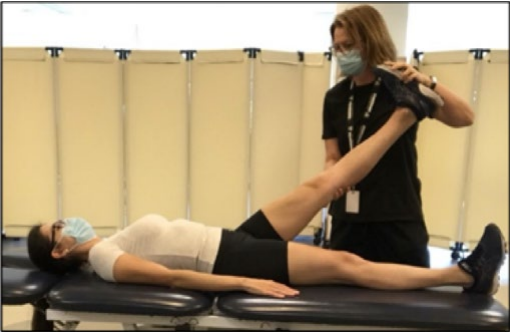 If the participant does not have radicular symptoms during the Passive Straight Leg Raise, then the tester places an inclinometer over the anterior tibial shaft, passively dorsiflexes the ankle, and lifts the leg off table as far as it goes while keeping the knee extended. If the participant has a positive Straight Leg Raise then the tightness of the posterior lower extremity muscles is not recorded.	Positive straight leg raise—Radicular symptoms in buttock/leg on tested side Positive crossed straight leg raise—Radicular symptoms in buttock/leg on the contralateral side For the tightness of posterior lower extremity muscles, the tester records the lower extremity flexion angle at the soft end feel in degrees
Special tests and hip mobility test		
Beighton Score [16] (6)	To test for generalized hypermobility, the participant is standing, and the tester assesses the ability to: Passively hyperextend 5th MCP beyond 90°^a^ Passively appose thumb to flexor aspect of forearm^a^ Passively hyperextend elbow beyond 10°^a^ Hyperextend knee beyond 5°^a^ Touch palms to the floor during flexion of the trunk with knees extended.	Record 1 point for each motion able to complete, for a total of 9 points possible Positive (score of 4–9) Negative (score of 0–3)
Hip Scour^a^ [17] (10)	Participant supine, tester passively flexes the hip and knee to full flexion and axially loads the femur. Tester moves the femur in an arc toward maximal adduction and internal rotation. 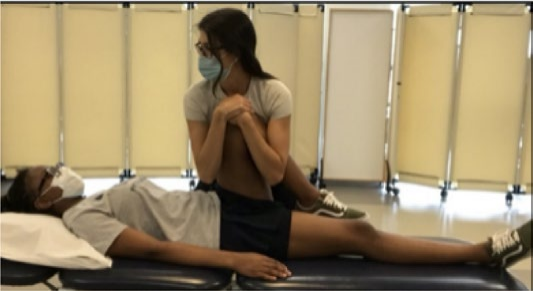	Positive—Provocation of lateral/posterior hip/groin symptoms
Active Straight Leg Raise^a^ [18, 19] (15)	Participant supine, raises straight leg ~5 cm off the table. If pelvic girdle pain or weakness is reported, tester applies bilateral pressure to the pelvis in the lateral to medial direction. 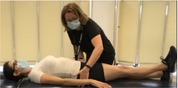	Positive—Pain or weakness is reduced or eliminated with the compression
Hip Internal Rotation Range of Motion^a^ [20, 21] (20)	Participant prone, knee flexed to 90°. Tester positions inclinometer 1–2 in. proximal to the medial malleolus on the medial distal tibia, stabilizes pelvis, and holds the distal tibia. Participant relaxes and tester moves the hip passively through internal rotation. 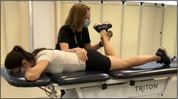	Hip internal rotation angle at the soft end feel in degrees
Tests of muscle function		
Hip Abduction Strength with HHD^a^ [22, 23, 24] (11)	Participant supine, feet beyond table edge. Tester positions the handheld dynamometer (HHD) just proximal to the superior edge of the lateral malleolus and participant pushes testing leg outward for 3–5 s as strong as they possibly can. Three trials are performed on each side with 5 s of rest in between. 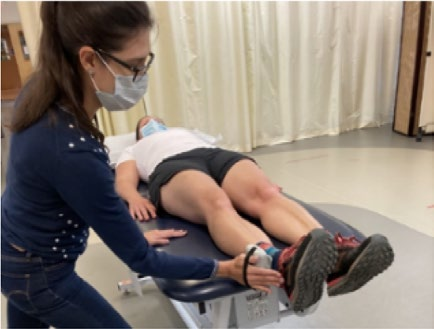	Force output in kilograms. Value is divided by body mass to represent force output as a percentage of body mass.
Hip Extension Strength with HHD^a^ [25] (21)	Participant prone with the knee bent on testing side. Tester positions HHD just proximal to popliteal space and participant pushes testing leg upwards for 3–5 s as strong as they possibly can. Three trials are performed on each side with 5 s of rest in between. 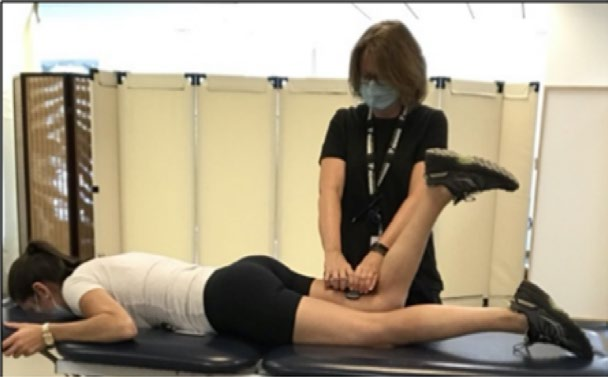	Force output in kilograms. Value is divided by body mass to represent force output as a percentage of body mass.
Hip External Rotation Strength with HHD^a^ [26] (22)	Participant prone, knee flexed to 90° on testing side and hip neutral. Tester positions HHD just proximal to the superior edge of the medial malleolus while stabilizing the pelvis and participant pushes testing leg outwards for 3–5 s as strong as they possibly can. Three trials are performed on each side with 5 s of rest in between. 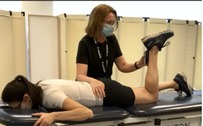	Force output in kilograms. Value is divided by body mass to represent force output as a percentage of body mass.
Quadriceps Strength [27] Option 1: Using the electronic dynamometer (Biodex) Option 2: Stair Climbing [28, 29] (23)	**Quadriceps Strength using the Biodex** ^a^: Participant sitting with testing knee flexed to ~70°. Tester positions the Biodex force pad on anterior shin just superior to intermalleolar line, and the axis of rotation is aligned with the femoral lateral epicondyle. The participant performs two warm‐ups and at least 3 trials of their maximum isometric knee extension on each side. Each trial is about 5 s with 30 s rest in between. *This test was replaced by Option 2 midway through study implementation due to the length of time required to complete it*. 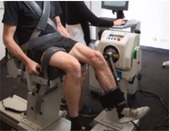 **Stair Climbing**: Participant ascends of one flight of stairs (11 steps with standard height of 17 cm) using a unilateral handrail for safety purposes and an assistive device as appropriate; the participant ascends the steps as fast as they can without compromising safety.	Option 1: Force output (Newton‐meter) Option 2: Time to go up the flight of stairs in seconds.
Active Sit‐up [30, 31] (31)	Participant supine, knees flexed to 90° and soles of feet flat on the table. Participant reaches both hands to touch kneecaps. 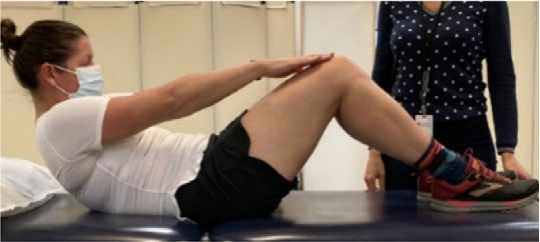	Number of seconds the position was held (maximum of 120 s)
Tests of Sacroiliac Joint (SIJ) Dysfunction [32, 33]		
Distraction (12)	Participant supine, tester applies bilateral posterolateral pressure on both ASIS. 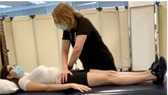	Positive—Pain in SIJ. Pain may be on the left side, right side, or bilateral.
Thigh Thrust^a^ (13)	Participant supine, tester flexes testing hip to 90°, rolls participant toward tester, stabilizes sacrum over tester's hand, and rolls participant back to supine. Tester applies downward axial pressure through the knee into the femur while maintaining testing hip at 90° of flexion. 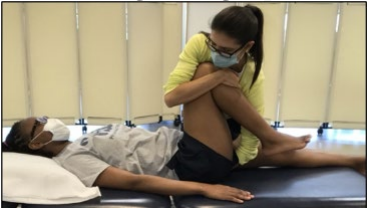	Positive—Pain in SIJ.
Gaenslen's^a^ (14)	Participant supine with the testing leg hanging off the table and the other hip fully flexed while participant grasps the knee of the fully flexed leg. The tester places downward pressure on the extended leg to move it further into extension while putting pressure on the flexed leg to push it further into flexion. The attempt is to rotate one innominate posteriorly and the other anteriorly. 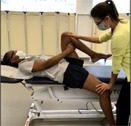	Positive—Pain in SIJ.
Compression^a^ (16)	Participant side lying, the tester presses downward on the ilium in a lateral to medial direction to apply a compressive force to the ipsilateral SI joint. 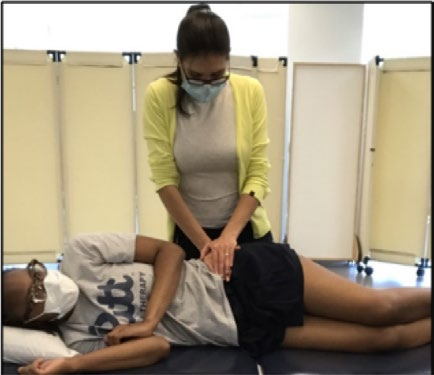	Positive—Pain in SIJ.
Sacral Thrust (17)	Participant prone, tester applies pressure in posterior to anterior direction on the apex of the sacral curve. 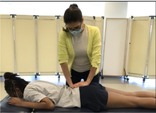	Positive—Pain in SIJ. Pain may be on the left side, right side, or bilateral.
Tests of spine mobility		
Lumbar Segmental Mobility [34, 35] (18)	Participant prone, the tester applies posterior to anterior pressure on lumbar spine, springing on the spinous process of each lumbar segment.	Mobility is recorded as painful, hypomobile, hypermobile, or normal for each lumbar segment.
Prone Instability^b^ [36] (19)	This test is only performed on participants with positive findings for pain during the lumbar segmental mobility test. Participant continues in prone and slides down on the table so that the pelvis is over the edge of the table and feet on the floor. Tester applies posterior to anterior pressure to painful lumbar spinous processes. Participant is then asked to lift the feet off the floor and tester applies the same pressure on spinous processes. 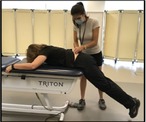	Positive—Pain over the spinal process experienced during the lumbar segmental mobility test decreases with the contraction of the muscles required to lift the legs and repeated posterior to anterior pressure on the tested segment.
Trunk Range of Motion (28) and Aberrant Motion (24)	Participant standing, performs a couple of trunk flexion and extension movements to assess Aberrant Motion and then 2 repetitions of full trunk flexion and extension to obtain goniometric measures. Test is done at participant self‐selected speed to maximize range of motion (ROM). The tester measures flexion and extension ROM in degrees (°) with the inclinometer over the spinous process of T12 (total ROM) during the 1st repetition and over S1 during the 2nd repetition (sacral ROM). While the participant performs lumbar flexion (going into flexion and returning from flexion) without the goniometer, the tester identifies painful arc, Gower Sign, reversal of lumbopelvic rhythm, and deviation from the sagittal plane and/or instability catch. These abnormalities are recorded.	Total ROM: movement recorded by inclinometer over T12. Sacral ROM: movement recorded by inclinometer over S1. Lumbar ROM: calculated by movement recorded over T12 minus S1. For Aberrant Motion, the tester records the following observations: Painful arc: Symptoms at a point during the motion that were absent before or after this point. Gower sign: Pushing on the thighs with the hands during the return from the flexed to the erect position. Reversal of lumbopelvic rhythm: On initiation of flexion, participant keeps the lumbar spine straight while flexing the hips first; on return from flexion, bends the knees and shifts the pelvis anteriorly before returning to the erect position. Deviation or “catch”: movement deviates from the primary plane of movement more than a few degrees.
Lumbar Quadrant^a^ [37, 38] (32)	Participant standing, extends, side bends and rotates the lumbar spine to one side. Tester gently guides participant through motion with one hand on the lumbar spine and the other on the shoulder. 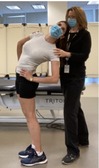	Positive is the presence of pain in back, leg, or back and leg
Repeated Movement Testing^a^ [37, 38, 39] (33)	Participant standing, performs 10 repetitions of lumbar flexion, extension, right‐side glide, and left‐side glide. If peripheralization is present during standing repeated flexion or side gliding, but no directional preference is observed, the participant lies prone and performs 10 repetitions of prone lying lumbar extension with or without side gliding. If peripheralization is present during standing repeated extension, but no directional preference is observed, the participant lies supine and performs lumbar flexion by bringing the knees close to the chest.	The tester records the presence of: Peripheralization (symptoms expand away from the center of the lower back during movement). Centralization (symptoms shrink toward the center of the lower back during movement). Directional preference (movement produces centralization or decreases pain intensity) during repeated movement tests.
Tests of functional performance		
Gait Speed—4‐m Walk [40, 41] (25)	Participant standing, walks 4 m at their self‐selected pace. Test is repeated twice.	Seconds elapsed from participant stepping over the starting line to participant's first foot crossing the finish line. The fastest trial is recorded.
5 Times Sit‐to‐stand [42, 43] (26)	Participant sitting, stands up and sits down in a 43 cm chair 5 times with arms crossed over the chest as fast as safely as possible.	Seconds elapsed from 1st stand to 5th stand
Standing Balance—Both legs [40, 44] (27)	Standing on both feet, participant is asked to maintain balance during 10 s without moving feet or grabbing for support during a sequence of three standing positions. Participant progresses to the next position only if able to balance during 10 s during less demanding conditions. Successful completion of each test is the ability to maintain balance in each testing condition for 10 s. 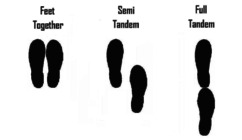	The test is scored from 0 (inability to perform the task) to 4 points (best test performance) by adding the points below: 1 point: hold side‐by‐side stand for 10 s. 1 point: hold semi‐tandem stand for 10 s. 1 point: hold tandem stand for 3–9.99 s. 2 points: hold tandem stand for 10 s. Total score of 4 points represents good performance. Total score from 0 to 3 points represents poor performance.
Standing Balance—Single‐leg [45] (28)	Participant standing, is asked to maintain balance on one foot (self‐selected) during 60 s without touching the other foot to the floor, moving feet, or grabbing for support.	Record the time the participant is able to balance on one foot (maximum of 60) in seconds.
2‐Minute Walk [46, 47, 48] (29)	Participant walks as fast and safely as possible for 2 min on a walking track.	Distance walked for 2 min (in meters)
Postural Lifting Strategy [41, 49] (30)	Participant standing, lifts a weighted box 4 times from the floor to 75 cm high table and back to the floor. Test ends once the box touches the Table 4 times or 20 s is reached (whichever comes first). Score is from 0 to 5. Zero represents good performance whereas 5 represents poor performance. 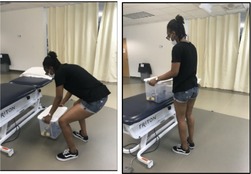	Score is a sum of qualitative points (up to 5): Spine neutral: ○ 0—keeps neutral spine ○ 1—unable to maintain neutral spine Flexion dominance: 0 –more hip/knee then pelvic/lumbar flexion 1 –more pelvic/lumbar then hip/knee flexion Base of support: ○ 0—wide base of support ○ 1—narrow base of support Aberrant movement: 0—no aberrant movement 1—aberrant movement Box proximity to the body: 0—box kept close to body/trunk 1—box not kept close to body/trunk
Quantitative sensory tests [50, 51]		
Pain Pressure Threshold (34)	Participant sitting on a chair, tester presses the probe of the algometer into the mid‐belly of upper trapezius and manually increases pressure by 0.5 kg per second until participant reports that the pressure sensation is painful. Repeat 3 trials with 60 s of rest in between. Participant then lies prone and the tester repeats the same procedures in the paravertebral lumbar area with the most pain. 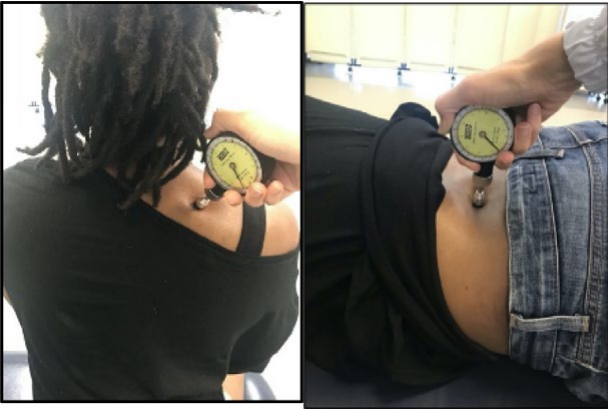	Record algometer value when pressure turns to pain (kilograms/square centimeter).
Pain Temporal Summation (35)	Participant is sitting with forearm resting on the examination table. The dominant volar forearm is pointed upwards. Tester uses Neuropen to apply 10 identical pinpricks to the area at a rate of 1 pinprick per second. Repeat 3 trials with 60 s of rest in between. Participant then lies prone and the tester repeats the same procedures in the paravertebral lumbar area with most pain. 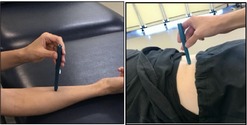	Record the pain intensity on a scale from 0 (no pain) to 10 (worst pain imaginable) of 1st prick and last prick. Lingering pain intensity on a scale from 0 to 10, 15, and 30 s after pricks.
Conditioned Pain Modulation (36)	Participant sits on a chair. While the participant places a hand up to the wrist in cold‐water tank of 5°C circulating water, the tester presses the probe of the algometer into the mid‐belly of upper trapezius and manually increases pressure by 0.5 kg per second until participant reports that pressure sensation is painful. The algometer tests on the trapezius contralateral to the hand immersed in cold water. Repeat the procedure twice with 120 s of rest in between. 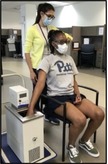	Record pressure intensity when pressure turns to pain (kilograms/square centimeter) while the opposite hand is immersed in the cold water.
Cold Pain Tolerance (37)	Participant sits on a chair and immerses the hand up to the wrist in a cold‐water tank of 5°C circulating water until pain tolerance is reached or up to a maximum time of 3 min (whichever comes first). 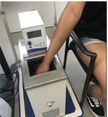	Record the number of seconds tolerated (maximum of 180 s) and the intensity of hand pain provoked by cold water immersion on a scale from 0 to 10 immediately, 30 s, 1 min, and 2 min after removing the hand from the water.

Abbreviations: HHD, hand‐held dynamometer; MCP, metacarpal phalangeal joint; ROM, range of motion.

^a^ Tests were done bilaterally.

^b^ Performed only if the pain is provoked during Lumbar Segmental Mobility Test.

The study used a customized electronic data capture system that enabled the testers to directly enter the physical test results using mobile tablets. The system was programmed to guide the testers on the sequence of tests and was designed with real‐time validity checks for acceptable values and prompts for outliers or missing values. The electronic data collection system also kept the times when each test's results were registered, and these data were used to calculate testing time. The time of each test corresponded to the time when the tester opened the system screen for a new test until the time when the final value of the test was submitted. Thus, the test time includes instructions provided by the tester, participant setup, practice trial (if applicable), test execution, and data recording/submission.

Prior to starting the physical tests, participants were screened to assess safety in the context of co‐existing medical conditions that could contraindicate testing performance. The electronic data capture system recorded the safety assessments and was programmed to automatically remove the tests for participants who had contraindications. Standardized elements of the safety screening are described below: [15, 52]

- Presence of extreme blood pressure or hypertensive crisis (systolic ≥ 180 and/or diastolic ≥ 120 mmHg). This finding excluded the participant from all physical tests and triggered immediate referral to care or, if symptomatic, emergency assistance.
- Hypertension with systolic blood pressure ≥ 150 and < 180, diastolic blood pressure ≥ 100 and < 120 mmHg, medical history (e.g., unstable cardiac disease, severe pulmonary hypertension, acute infections), or self‐reported symptoms (e.g., dizziness, blackouts, chest pain, shakiness). These findings excluded participants from tests that required maximum or submaximal effort (i.e., Muscle Strength Tests with a dynamometer, Stair Climbing Test, Prone Instability test, and Active Sit‐Up Tests) or could increase blood pressure (i.e., hand immersion in the cold‐water tank). When applicable, these participants were instructed to discuss findings with their physician.
- Circulatory or sensory conditions affecting the hands (e.g., Raynaud's Syndrome, Complex Regional Pain Disorder) or related symptoms (e.g., painful, pale, reddish, bluish fingers). These findings excluded hand immersion in the cold‐water tank during the Conditioned Pain Modulation and Cold Pain Tolerance tests.

In addition to the pre‐defined safety screening, the testers could elect not to administer tests based on their clinical judgment, and participants could refuse to partake in physical tests. The testers recorded the reasons for not performing tests and adverse events (AEs) related to test performance.

### 2.3 Changes in Testing Protocol

There were two changes in testing protocol during study implementation. The first was an unintentional programming error in the electronic data capture system at the beginning of the study that screened out myotome tests for participants with high blood pressure. This error was identified during a fidelity check (after 158 participants had completed the assessment) and the clinical team approved the myotome tests for all participants going forward, except in the presence of extreme blood pressure. The second change was related to the protocol for the quadriceps strength testing. At the start of the study, the team used a custom frame that enabled rapid quadriceps strength testing with the participant in a prone position to prevent lumbar flexion. Due to equipment failure, this test did not provide consistent values, and we switched to using an electronic dynamometer (Biodex Medical System, Shirley, NY) for testing. However, the electronic dynamometer test added at least 30 min to the already long study visit, resulting in a high rate of participants refusing the test due to time restriction. Thus, this test was replaced with the Stair Climbing Test, a more efficient functional test widely used to assess the strength and power of the quadriceps along with the other muscles of the lower extremities [28].

### 2.4 Data Analysis

The descriptive statistics for the demographic and biomedical characteristics of participants who performed all tests and those who did not perform one or more tests are provided to visually assess potential factors influencing participation in the physical tests. We elected to discuss factors for which the differences between subgroups that completed all tests or did not complete all tests were considered clinically relevant or were > 5%. Feasibility of the physical tests was assessed by (1) computing the mean and standard deviation (SD) for the time to complete each test, (2) calculating the frequencies of tests not done, (3) describing aggregate reasons for tests not done, and (4) describing AEs related to testing performance. Lastly, we provide descriptive statistics of the physical tests for the entire study sample and subgroups based on sex at birth and age (male/female and age < 60/≥ 60) to serve as reference values for individuals with cLBP.

## 3 Results

Information about recruitment and enrollment was provided elsewhere in this issue in the article describing our cohort's clinical characteristics [53]. Table 2 characterizes the sample and highlights that from the 1007 participants enrolled in the study, those who did not perform one or more tests were 9.5 years older, less educated, and more severely disabled than those who performed all tests. Further, those who did not perform one or more tests had higher BMIs, more frequent previous back surgeries, and more than 5 years of LBP. Likely due to older age, those who missed one or more tests had more comorbidities compared to individuals who performed all tests and were also more likely to be retired and not employed.

**TABLE 2:** Select demographic and biomedical characteristics for participants who performed all tests and those who did not perform one or more tests.

*N* = 1007	Performed all tests, *N* = 389	Not performed one or more tests, *N* = 618
Age, mean ± SD, years	52.5 ± 16.5	63.0 ± 15.1
Sex at birth,^a^ *N* (%)		
Female	242 (62)	358 (58)
Male	146 (38)	260 (42)
Race, *N* (%)^b^		
White	301 (77)	458 (74)
African American	59 (15)	122 (20)
Other	14 (4)	9 (1)
More than one race	6 (2)	20 (3)
Missing	9 (2)	9 (1)
Ethnicity, *N* (%)		
Hispanic or Latino	12 (3)	14 (2)
Not Hispanic or Latino	360 (93)	543 (88)
Missing	17 (4)	61 (10)
Education, *N* (%)		
Less than Secondary School	18 (5)	44 (7)
Secondary School Degree	83 (21)	164 (27)
Associate's or Technical Degree	51 (13)	117 (19)
College Degree	120 (31)	150 (24)
Postgraduate	117 (30)	142 (23)
Relationship status, *N* (%)		
Married/have partner	222 (57)	319 (52)
Divorced/have no partner	166 (43)	299 (48)
Missing	1 (0)	0 (0)
Employment status, *N* (%)		
Full‐time employment	164 (42)	116 (19)
Part‐time employment	63 (16)	89 (14)
Student	10 (3)	5 (1)
Retired	93 (24)	285 (46)
Choose not to work	5 (1)	5 (1)
Not employed	54 (14)	118 (19)
How long is back pain ongoing, *N* (%)		
3–6 months	12 (2)	10 (2)
6 months to 1 year	25 (6)	44 (7)
1–5 years	134 (34)	171 (28)
> 5 years	218 (56)	393 (64)
Previous low back surgery, *N* (%)		
No	328 (84)	432 (70)
Yes	61 (16)	186 (30)
BMI (kg/m^2^), mean ± SD^c^	30.2 ± 6.7	32.3 ± 7.9
Comorbidities, mean ± SD^c^, ^d^	2.6 ± 2.1	3.7 ± 2.3
Low back pain intensity in the last week (0–10)	5.2 ± 2.3	5.6 ± 2.2
Oswestry Disability Index (0–100)^c^	25.7 ± 12.6	34.4 ± 16.0

^a^ One participant reported sex at birth as intersex and was therefore not included in the descriptive statistics for male/female. Intersex was not reported separately due to identification potential.

^b^ Other: Asian, American Indian or Alaskan Native, and Hawaiian or Pacific Islander races were combined due to small cell numbers and identification potential.

^c^ *N* = 1006 because a participant had to leave the assessment before completing these surveys or the physical test.

^d^ Based on comorbidities diagnosed in the past or experienced currently, including cancer, anxiety, depression, osteoarthritis, diabetes, thyroid disease, osteoporosis, multiple sclerosis, spinal cord injury, balance problems/falls and chronic overlapping pain conditions (migraine or chronic headache, irritable bowel syndrome, temporomandibular joint dysfunction, interstitial cystitis/irritable bladder, fibromyalgia, chronic fatigue, and for women only, endometriosis and vulvodynia).

### 3.1 Changes in Testing Protocol

The changes in testing protocol during study implementation resulted in 33 participants not performing the myotome tests due to a programming error in the electronic data capture system (Table 3). Due to changes in the quadriceps strength testing protocol, the first 416 participants underwent testing using the custom frame protocol; however, these data were excluded due to inconsistent results. The following 107 participants were not tested while awaiting protocol change approval. The next 101 participants were assigned the Biodex protocol, of which 58 were actually tested. Finally, 300 out of the last 382 participants were tested using the Stair Climbing protocol (Tables 3 and 4).

**TABLE 3:** Test duration, percentage of tests not done, and the reasons for tests not completing the tests.

*N* = 1006 unless stated	Test duration^a^ (in seconds), mean ± SD	Tests not done (%)		
		Overall	Pre‐defined screened out for safety reason	At tester's discretion or participant's refusal
Neurological examination				
Sensation (L4, L5, and S1 dermatomes)^d^	177 ± 126	0.9%	0.7%	0.2%
Reflexes (patellar, medial hamstring, and Achilles DTRs and Babinski)^d^	107 ± 51	1.0%	0.7%	0.3%
Myotome Testing^b^ (hip flexion, knee extension, ankle dorsiflexion, great toe extension, hamstrings)^d^	145 ± 56	6.1%	4.7%	1.4%
Myotome Testing—Single‐leg Calf Raise	119 ± 56	4.9%	3.4%	1.5%
Slump Test	101 ± 53	1.4%	0.7%	0.7%
Passive Straight Leg Raise and Crossed Straight Leg Raise Tests (include measuring tightness of posterior lower extremity muscles).	169 ± 81	2.0%	0.7%	1.3%
Special tests and hip mobility test				
Beighton Score	154 ± 70	0.7%	0.7%	0.0%
Hip Scour	99 ± 46	6.9%	0.7%	6.2%
Active Straight Leg Raise	61 ± 51	2.5%	0.7%	1.8%
Hip Internal Rotation Range of Motion	120 ± 74	4.2%	0.7%	3.5%
Tests of muscle function^c^				
Hip Abduction Strength with HHD	407 ± 114	21.3%	19.0%	2.3%
Hip Extension Strength with HHD	251 ± 76	25.8%	19.0%	6.9%
Hip External Rotation Strength with HHD	216 ± 75	24.2%	19.0%	5.3%
Quadriceps Strength with Biodex (*N* = 101)	1190 ± 776	57.4%	15.0%	42.4%
Stair Climbing (*N* = 382)	270 ± 187	21.5%	17.5%	3.9%
Active Sit‐Up	199 ± 64	26.8%	18.4%	8.5%
Tests of sacroiliac joint dysfunction				
Distraction	85 ± 83	4.3%	0.7%	3.6%
Thigh Thrust	99 ± 51	9.3%	0.7%	8.7%
Gaenslen's	101 ± 48	9.4%	0.7%	8.8%
Compression	80 ± 57	3.3%	0.7%	2.6%
Sacral Thrust	89 ± 109	4.3%	0.7%	3.6%
Tests of spine mobility				
Lumbar Segmental Mobility (pain, hypomobility and hypermobility, L1 to L5)	153 ± 95	9.9%	5.8%	4.2%
Prone Instability^e^	82 ± 125	21.3%	12.7%	8.6%
Trunk Range of Motion (include Aberrant Motion test).	307 ± 101	4.0%	0.7%	3.3%
Lumbar Quadrant	143 ± 98	7.2%	0.7%	6.5%
Repeated Movement Testing (flexion, extension, and side glide)^d^	273 ± 11	4.7%	0.7%	4.0%
Tests of functional performance				
Gait Speed; 4‐m Walk	164 ± 100	1.9%	0.7%	1.2%
5 Times Sit‐to‐stand	122 ± 47	10.7%	4.8%	6.0%
Standing Balance, both legs	197 ± 100	3.9%	0.7%	3.2%
Standing Balance, single‐leg	74 ± 25	8.9%	0.7%	8.2%
2‐Minute Walk Test	166 ± 81	7.4%	4.9%	2.5%
Postural Lifting Strategy	390 ± 123	13.6%	5.5%	8.2%
Quantitative sensory tests^f^				
Pain Pressure Threshold	228 ± 134	4.3%	0.7%	3.6%
Pain Temporal Summation	595 ± 214	2.8%	0.7%	2.1%
Conditioned Pain Modulation	638 ± 183	35.6%	30.8%	4.8%
Cold Pain Tolerance	600 ± 144	34.6%	30.7%	3.9%
Summary feasibility				
Test protocol total time (seconds)	7825 ± 1682	—	—	—
Test protocol total time (minutes)	130 ± 28	—	—	—
Average—Tests not done	—	8.9%	3.0%	5.9%

Abbreviation: HHD, hand‐held dynamometer.

^a^ The time for each test includes the instructions, participant setup, test execution, and data recording.

^b^ Forty participants (4%) were unintentionally screened out of the myotome testing.

^c^ The values for quadriceps strength using the customized frame are not reported because equipment malfunction resulted in inconsistent test results.

^d^ The components of this test were combined for estimating time (e.g., for sensation, the score combines testing the dermatomes of L4, L5, and S1). The percentages of tests not done represent the component of the test with the highest percentage of skipped tests.

^e^ Test was done only if the lumbar segmental mobility had a painful segment.

^f^ The values of these tests are included elsewhere in this issue [54].

**TABLE 4:** Descriptive statistics of clinical examination and performance‐based tests of participants.

	All	Male^a^	Female^a^	Age < 60	Age ≥ 60
	*N* = 999^b^	*N* = 401	*N* = 596	*N* = 424	*N* = 575
Neurological examination					
Sensation—L4 dermatome					
Normal, *N* (%)	907 (91)	360 (90)	546 (91)	384 (91)	523 (91)
Abnormal, *N* (%)	88 (9)	39 (10)	49 (8)	38 (9)	50 (9)
Sensation—L5 dermatome					
Normal, *N* (%)	868 (87)	340 (85)	527 (88)	368 (87)	500 (87)
Abnormal, *N* (%)	127 (13)	59 (15)	68 (11)	54 (13)	73 (13)
Sensation—S1 dermatome					
Normal, *N* (%)	869 (87)	349 (87)	519 (87)	365 (86)	504 (88)
Abnormal, *N* (%)	126 (13)	50 (12)	76 (13)	57 (13)	69 (12)
Deep Tendon Reflex – Patellar (L4)					
Normal, *N* (%)	549 (55)	209 (52)	340 (57)	255 (60)	294 (51)
Abnormal, *N* (%)	309 (31)	140 (35)	168 (28)	112 (26)	197 (34)
Deep Tendon Reflex—Medial Hamstrings (L5)					
Normal, *N* (%)	609 (61)	248 (62)	361 (60)	281 (66)	328 (57)
Abnormal, *N* (%)	274 (27)	115 (29)	158 (26)	93 (22)	181 (31)
Deep Tendon Reflex—Achilles (S1)					
Normal, *N* (%)	609 (61)	225 (56)	384 (64)	297 (70)	312 (54)
Abnormal, *N* (%)	289 (29)	134 (33)	154 (26)	95 (22)	194 (34)
Babinski Reflex					
Normal, *N* (%)	978 (98)	392 (98)	585 (98)	413 (97)	565 (98)
Abnormal, *N* (%)	15 (2)	6 (1)	9 (2)	8 (2)	7 (1)
Myotome Testing—Hip Flexion (L2/L3)					
Normal, *N* (%)	546 (55)	266 (66)	279 (47)	269 (63)	277 (48)
Abnormal, *N* (%)	407 (41)	122 (30)	285 (48)	129 (30)	278 (48)
Myotome Testing—Knee Extension (L3/L4)					
Normal, *N* (%)	844 (84)	357 (89)	486 (81)	357 (84)	487 (85)
Abnormal, *N* (%)	109 (11)	30 (7)	79 (13)	41 (10)	68 (12)
Myotome Testing—Ankle Dorsiflexion (L4)					
Normal, *N* (%)	835 (83)	347 (87)	487 (82)	361 (85)	474 (82)
Abnormal, *N* (%)	117 (12)	41 (10)	76 (13)	36 (8)	81 (14)
Myotome Testing—Great Toe Extension (L5)					
Normal, *N* (%)	788 (79)	323 (81)	464 (78)	355 (84)	433 (75)
Abnormal, *N* (%)	157 (16)	63 (16)	94 (16)	40 (9)	117 (20)
Myotome Testing—Knee Flexion (S1)					
Normal, *N* (%)	788 (79)	341 (85)	446 (75)	343 (81)	445 (77)
Abnormal, *N* (%)	165 (17)	46 (11)	119 (20)	55 (13)	110 (19)
Myotome Testing—Single‐Leg Calf Raise (S1)					
Normal, *N* (%)	660 (66)	265 (66)	394 (66)	329 (78)	331 (58)
Abnormal, *N* (%)	300 (30)	126 (31)	174 (29)	76 (18)	224 (39)
Slump Test					
Negative *N* (%)	846 (85)	351 (88)	494 (83)	344 (81)	502 (87)
Positive, *N* (%)	122 (12)	45 (11)	77 (13)	68 (16)	54 (9)
Passive Straight Leg Raise					
Positive on the side of elevated leg, *N* (%)	131 (13)	43 (11)	88 (15)	67 (16)	64 (11)
Positive on the contralateral side, *N* (%)	10 (1)	2 (0)	8 (1)	3 (1)	7 (1)
If negative, tightness of posterior leg muscles (°)					
Left, mean ± SD (*N* = 892)	69.8 ± 14.6	63.3 ± 12.2	74.4 ± 14.4	71.4 ± 14.6	68.7 ± 14.5
Right, mean ± SD (*N* = 895)	69.3 ± 14.5	63.0 ± 12.5	73.7 ± 14.3	71.1 ± 14.4	68.0 ± 14.5
Special tests and hip mobility test					
Beighton Score					
Positive, *N* (%)	36 (4)	10 (2)	26 (4)	33 (8)	3 (1)
Negative, *N* (%)	962 (96)	391 (98)	570 (95)	390 (92)	572 (99)
Hip Scour Test					
Left, *N* positive (%)	350 (35)	128 (32)	222 (37)	166 (39)	184 (32)
Right, *N* positive (%)	308 (31)	107 (27)	201 (34)	145 (34)	335 (58)
Active Straight Leg Raise Test					
Left, *N* positive (%)	162 (16)	65 (16)	96 (16)	67 (16)	95 (17)
Right, *N* positive (%)	210 (21)	69 (17)	140 (23)	92 (22)	118 (21)
Hip Internal Rotation Mobility					
Left (°), mean ± SD (*N* = 959)	34.1 ± 11.7	28.2 ± 10.1	38.1 ± 11.1	36.8 ± 10.9	32.1 ± 12.0
Right (°), mean ± SD (*N* = 966)	33.6 ± 11.9	27.7 ± 9.9	37.6 ± 11.4	35.9 ± 11.1	31.9 ± 12.1
Tests of muscle function					
Hip Abduction Strength with HHD (% of body mass)					
Left, mean SD (*N* = 793)	10.1 ± 3.7	11.8 ± 4.0	8.9 ± 2.9	11.5 ± 3.8	8.9 ± 3.1
Right, mean SD (*N* = 796)	10.3 ± 3.7	12.1 ± 3.9	9.1 ± 2.9	11.8 ± 3.6	9.1 ± 3.3
Hip Extension Strength with HHD (% of body mass)					
Left, mean SD (*N* = 750)	13.5 ± 6.2	15.6 ± 7.0	12.1 ± 5.1	15.4 ± 6.5	11.9 ± 5.4
Right, mean SD (*N* = 756)	13.5 ± 6.5	16.0 ± 7.2	11.9 ± 5.3	15.3 ± 6.7	12.0 ± 5.8
Hip External Rotation Strength with HHD (% of body mass)					
Left, mean SD (*N* = 763)	9.3 ± 3.4	11.3 ± 3.7	7.9 ± 2.4	10.3 ± 3.5	8.4 ± 3.1
Right, mean SD (*N* = 766)	9.7 ± 3.6	12.0 ± 3.9	8.2 ± 2.5	10.6 ± 3.7	8.9 ± 3.5
Quadriceps Strength using Biodex (Nm)					
Left, mean ± SD (*N* = 58)	140.6 ± 54.8	180.6 ± 56.3	117.9 ± 39.2	159.5 ± 63.6	120.3 ± 34.4
Right, mean ± SD (*N* = 56)	134.9 ± 48.8	171.4 ± 49.3	113.1 ± 33.3	150.6 ± 53.2	116.8 ± 36.3
Stair Climbing (sec), mean ± SD (*N* = 300)	7.3 ± 4.0	7.1 ± 4	7.5 ± 4.1	5.9 ± 3.2	8.4 ± 4.3
Active Sit‐Up (sec), mean ± SD (*N* = 789)	23.8 ± 34.2	32.5 ± 39.7	18.0 ± 28.5	27.5 ± 34.1	20.8 ± 34.0
Tests of Sacroiliac Joint (SIJ) Dysfunction (positive)					
SIJ Distraction					
Bilateral Pain, *N* (%)	71 (7)	21 (5)	49 (8)	44 (10)	27 (5)
Left pain, *N* (%)	27 (3)	13 (3)	14 (2)	8 (2)	19 (3)
Right pain, *N* (%)	50 (5)	17 (4)	33 (6)	18 (4)	32 (6)
Thigh Thrust					
Left, *N* (%)	227 (23)	70 (17)	157 (26)	106 (25)	121 (21)
Right, *N* (%)	231 (23)	62 (15)	169 (28)	108 (25)	123 (21)
Gaenslen's Test					
Left, *N* (%)	122 (12)	45 (11)	77 (13)	53 (13)	69 (12)
Right, *N* (%)	146 (15)	48 (12)	98 (16)	67 (16)	79 (14)
SIJ Compression					
Left, *N* (%)	73 (7)	22 (5)	51 (9)	30 (7)	43 (7)
Right, *N* (%)	93 (9)	32 (8)	61 (10)	45 (11)	48 (8)
Sacral Thrust					
Bilateral pain, *N* (%)	137 (14)	44 (11)	92 (15)	72 (17)	65 (11)
Left pain, *N* (%)	71 (7)	26 (6)	45 (8)	31 (7)	40 (7)
Right pain, *N* (%)	69 (7)	19 (5)	50 (8)	32 (8)	37 (6)
Tests of Spine Mobility					
Lumbar Segment with Pain					
L1, *N* (%)	211 (21)	75 (19)	136 (23)	87 (20)	124 (22)
L2, *N* (%)	280 (28)	101 (25)	178 (30)	122 (29)	158 (27)
L3, *N* (%)	353 (35)	137 (34)	215 (36)	158 (37)	195 (34)
L4, *N* (%)	422 (42)	159 (40)	262 (44)	202 (48)	220 (38)
L5, *N* (%)	446 (45)	156 (39)	289 (48)	229 (54)	217 (38)
Lumbar Segment Hypomobile					
L1, *N* (%)	298 (30)	165 (41)	133 (22)	76 (18)	222 (39)
L2, *N* (%)	307 (31)	169 (42)	138 (23)	80 (19)	227 (39)
L3, *N* (%)	348 (35)	190 (47)	158 (26)	97 (23)	251 (44)
L4, *N* (%)	405 (41)	211 (53)	194 (32)	119 (28)	286 (50)
L5, *N* (%)	435 (44)	225 (56)	210 (35)	135 (32)	300 (52)
Lumbar Segment Hypermobile					
L1, *N* (%)	49 (5)	10 (2)	39 (7)	42 (10)	7 (1)
L2, *N* (%)	52 (5)	10 (2)	42 (7)	42 (10)	10 (2)
L3, *N* (%)	56 (6)	12 (3)	44 (7)	46 (11)	10 (2)
L4, *N* (%)	51 (5)	10 (2)	41 (7)	44 (10)	7 (1)
L5, *N* (%)	48 (5)	10 (2)	38 (6)	43 (10)	5 (1)
Prone Instability, *N* positive (%)^c^	177 (18)	73 (18)	103 (17)	93 (22)	84 (15)
Trunk Range of Motion (°)					
Total flexion, mean ± SD, (*N* = 938)	86.4 ± 24.3	76.3 ± 20.5	93.2 ± 24.5	92.8 ± 25.0	81.5 ± 22.6
Total extension, mean ± SD, (*N* = 954)	19.5 ± 10.2	17.4 ± 9.2	20.8 ± 10.7	22.1 ± 11.9	17.6 ± 8.2
Sacral flexion, mean ± SD, (*N* = 947)	61.2 ± 19.6	55.3 ± 17.0	65.2 ± 20.3	60.5 ± 21.1	61.7 ± 18.3
Sacral extension, mean ± SD, (*N* = 954)	10.3 ± 7.4	9.5 ± 6.6	10.8 ± 7.8	10.5 ± 8.4	10.1 ± 6.6
Lumbar flexion, mean ± SD, (*N* = 951)	25.1 ± 13.9	21.1 ± 11.9	27.8 ± 14.6	32.1 ± 13.9	19.9 ± 11.5
Lumbar extension, mean ± SD, (*N* = 954)	9.2 ± 8.6	7.9 ± 7.2	10.0 ± 9.3	11.6 ± 10.0	7.4 ± 6.9
Lumbar Quadrant Positive					
Left, *N* (%)	604 (60)	229 (57)	374 (63)	268 (63)	336 (58)
Right, *N* (%)	597 (60)	236 (59)	360 (60)	271 (64)	326 (57)
Repeated Movement Testing					
Peripheralization, *N* (%)	108 (11)	35 (9)	73 (12)	41 (10)	67 (12)
Centralization, *N* (%)	52 (5)	17 (4)	34 (6)	25 (6)	27 (5)
Directional preference, *N* (%)	48 (5)	12 (3)	36 (6)	23 (5)	25 (4)
Aberrant Movement, *N* (%)	14 (1)	8 (2)	6 (1)	7 (2)	7 (1)
Tests of functional performance					
Gait Speed—4‐m Walk (m/s), mean ± SD, (*N* = 985)	0.9 ± 0.2	0.9 ± 0.2	0.9 ± 0.2	1.0 ± 0.2	0.9 ± 0.2
5 Times Sit‐to‐Stand Test (sec), mean ± SD, (*N* = 894)	13.4 ± 5.1	13.3 ± 5.4	13.4 ± 4.9	12.8 ± 5.6	13.8 ± 4.6
Standing Balance, both legs, (*N* = 981)					
Poor Performance, *N* (%)	165 (17)	93 (23)	72 (12)	33 (8)	132 (23)
Good Performance, *N* (%)	816 (82)	306 (76)	509 (85)	386 (91)	430 (75)
Standing Balance, single‐leg (sec), mean ± SD, (*N* = 888)	30.4 ± 24.4	27.6 ± 24.6	32.2 ± 24.2	42.4 ± 22.5	20.4 ± 21.3
2‐Minute Walk (m), mean ± SD, (*N* = 930)	165.8 ± 44.2	169.6 ± 48.7	163.2 ± 40.6	178.2 ± 42.8	156.8 ± 43.1
Postural Lifting Strategy (0–5), mean ± SD, (*N* = 866)	2.4 ± 1.2	2.4 ± 1.2	2.4 ± 1.2	2.3 ± 1.2	2.5 ± 1.2

^a^ One participant reported sex at birth as intersex and was therefore not included in the descriptive statistics for male/female.

^b^ The *N* used as the denominators for calculating percentages are in the top row, unless stated otherwise.

^c^ Test was done in 477 participants who had a painful segment during the lumbar segmental mobility test. However, the percentage of positive tests was calculated on the overall sample (*N* = 999).

### 3.2 Feasibility of the Physical Tests

The feasibility analysis included 1006 individuals because one participant had to leave the assessment visit before completing the safety screening for the physical tests. The overall testing protocol took, on average, 2 h (130 min). Splitting the assessment visit into two visits to minimize fatigue was needed for less than 1% of participants.

The longest tests were the Quadriceps Strength using the Biodex (20 min, or 1190 ± 776 s), Conditioned Pain Modulation (11 min, or 637 ± 183 s), Cold Pain Tolerance and Pain Temporal Summation (10 min for each test, or 600 s on average), Hip Abduction Strength (7 min or 407 ± 114 s), and Trunk Range of Motion (5 min, or 307 ± 101 s) (Table 3, 2nd column). The tests most frequently omitted were the Quadriceps Strength using the Biodex, Conditioned Pain Modulation, Cold Pain Tolerance, Active Sit‐Up, Prone Instability, Stair Climbing, and the battery of Hip Extension, Abduction, and External Rotation Strength. On the other hand, the tests less frequently omitted were the components of the neurological examination such as sensation, reflexes, Slump Passive Straight Leg Raise, Beighton Score, 4‐m Walk, Active Straight Leg Raise, Pain Temporal Summation, Standing Balance, and Compression for sacroiliac joint dysfunction (Table 3, 3rd column).

**FIGURE 1:**
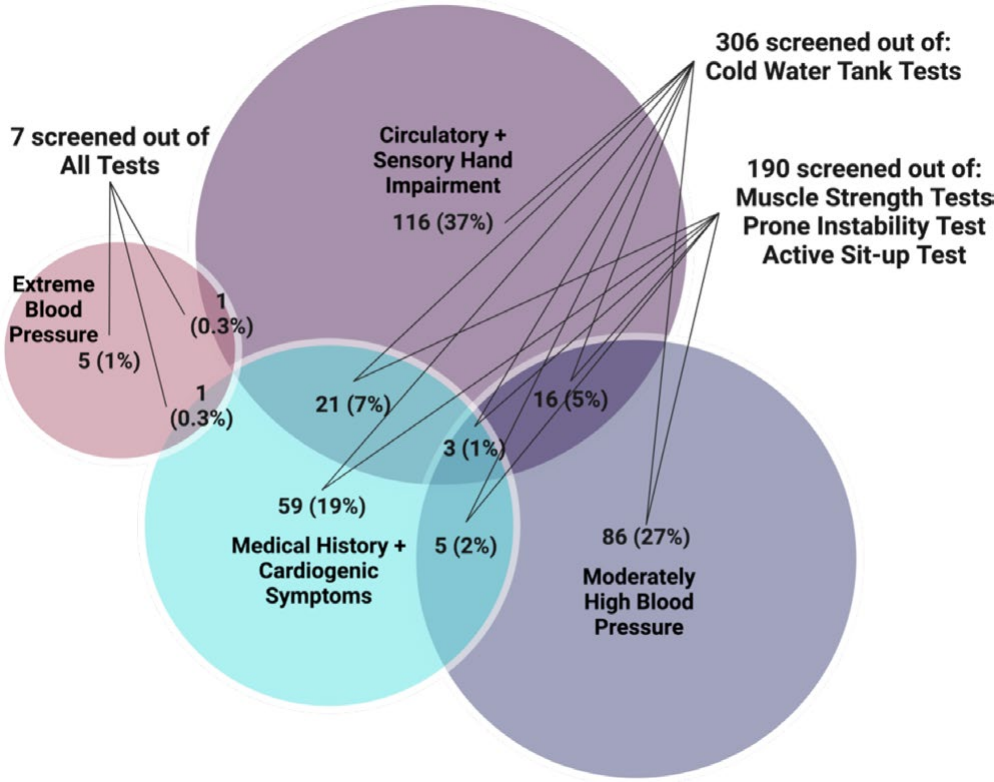
A total of 313 participants were screened out from one or more tests due to predefined safety screening for physical tests. *N* = 313 served as the denominator of the frequency calculations in this figure. Participants could be excluded from tests for more than one reason. There was a total of 361 reasons for being screened out and with the most frequent safety screening factor being circulatory and sensory hand impairment (*N* = 157), followed by hypertension (*N* = 110), medical history and/or cardiogenic symptoms (*N* = 89), and extreme blood pressure (*N* = 7).

In total, 8.9% of tests were not done, of which about a third were screened out during the pre‐defined safety screening, and two‐thirds due to the tester's clinical judgment or the participant's refusal (Table 3). For the former, a total of 313 participants (31%) were screened out from one or more tests. Seven participants were screened out due to extreme blood pressure, 116 due to circulatory or sensory hand impairment, 86 due to hypertension, 59 due to medical history or cardiogenic symptoms, and 45 due to two or more reasons (Figure 1).

The reasons for missing tests due to the tester's clinical judgment were wide‐ranging (data not shown). The most common being participant's inability to obtain the testing start position (e.g., Active Sit‐up and Single‐leg Balance); a hip prosthesis limiting test performance (e.g., Hip Scour and Hip Internal Rotation); heightened pain limiting pain‐provocation tests (e.g., Lumbar Quadrant); intolerance to the prone position; muscle weakness limiting standing from a chair unassisted or lifting a box from the floor; and poor balance or use of a wheelchair preventing safe performance of standing and walking tests. The participant's main reasons for testing refusal were severe back pain and time restriction. The latter mainly prevented the lengthy quadriceps strength test on the Biodex (Table 3, 4th and 5th columns).

There were four AEs related, or possibly related, to performing the physical tests (data not shown). Two were moderate exacerbations of LBP, one was new radiating pain to the buttock and back or the leg, and one was new groin pain. All AEs resolved without sequela.

### 3.3 Descriptive Statistics of the Physical Tests

Of the 1006 individuals who participated in the safety screening for the physical tests, 7 had extreme blood pressure and could not participate in any physical tests, resulting in 999 individuals in descriptive analysis. The prevalence of abnormal findings during neurological screening, for all participants combined, ranged from 9% to 13% for sensation, 27% to 31% for deep tendon reflexes, 11% to 41% for myotome testing, and 2% for the Babinski sign (Table 4). Special tests such as the Beighton Score, Hip Scour, Active Straight Leg Raise, and Aberrant Movement tests were positive in 4%, 33%, 19%, and 1% of the sample, respectively. The prevalence of positive tests for sacroiliac joint dysfunction for all participants ranged from 8% for the Compression Test to 28% for the Sacral Thrust Test.

The mobility tests for all individuals demonstrated an average of 34° of hip internal rotation and positive lumbar segment mobility test findings for pain on average for 34% of individuals, and for hypomobility in 36% of individuals. The prevalence of positive findings increased at the lower segments of the lumbar spine. For example, pain was positive in 21% of participants in L1 and 45% in L5, and hypomobility was positive in 30% of participants in L1 and in 44% in L5. Lumbar segment mobility was positive for hypermobility in 5% of participants and rates were consistent at each level. Of those with pain during segmental mobility, 18% had a positive Prone Instability Test. Additionally, across all individuals the average total trunk flexion was 86°, of which 25° happened in the lumbar spine and 61° in the pelvis or hip joints; whereas total extension was 19°, of which 9° was in the lumbar and 10° in the pelvis or hip joints.

Measures of muscle function for all participants demonstrated that the strength of the hip muscles ranged from 9.3% to 13.5% of body weight, the average quadriceps strength was 138 newton‐meter (Nm), and participants were able to maintain the active sit‐up position for about 29 s. Functional performance tests revealed an average self‐selected gait speed of 0.9 m/s, time to stand from a chair five times of 13.4 s, time to climb one set of stairs of 7.3 s, and distance walked in 2 min of 165 m.

Findings stratified by biological sex and age demonstrated that the older adults had a higher prevalence of abnormal deep tendon reflexes, lower prevalence of tests of neural tension (Slump and Passive Straight Leg Raise), and worse functional performance demonstrated by slow gait speed, slow time to stand from a chair, poor walking endurance, and poor balance compared to the younger group. In general, the older group and males had less joint mobility (e.g., Beighton Score, Hip Internal Rotation, Lumbar Segmental Mobility, and lumbar and sacral range of motion). Additionally, the older group and females had less muscle strength and endurance (e.g., Myotome Tests, Active Sit‐up Test, and tests of muscle strength using the dynamometer).

## 4 Discussion

This work assessed the feasibility of a comprehensive battery of physical tests and pain assessments germane to individuals with cLBP. The results indicate that individuals with cLBP were able to partake in the vast majority (91.1%) of all tests—with only four AEs, all of which resolved without sequelae, supporting the safety of this test battery. Participants who did not perform all tests tended to be older, obese, less educated, and experienced more disability and back pain for a longer time. While information on these demographic factors can help forecast potential missing data in future studies, we caution against using this information for potentially profiling participants and unintentionally triggering inequitable research and clinical care. This study also provides novel information on the tests' performance frequency, reasons for not being completed, duration, and descriptive results in individuals with cLBP. This comprehensive characterization provides reference values for research planning and prevailing values for comparison in clinical practice.

Among the 37 tests, 18 were done by > 95% of participants, 26 by > 90%, 28 by > 80%, 34 by > 70%, and only three tests by lower than 70%. The tests more frequently done (> 95%) were the neurological examination tests, the Active Straight Leg Raise, the 4‐m Walk, and the Pain Temporal Summation tests. Of note, while the data show the myotome tests were not done 4.9%–6.1% of the time, the tolerance for these tests was excellent. This higher‐than‐expected rate of skipping myotome tests was because, at the beginning of the study, the data entry system was unintentionally programmed to screen out these tests. After fixing this error, the myotome tests screened out were mainly due to heightened pain. This observation highlights the importance of frequent data quality control to prevent missing data.

The Quadriceps Strength Test using the Biodex was by far the most frequently omitted test, with 57% of tests not done: 15% screened out for safety reasons and 42% refused due to test length. After this test was attempted in about 200 participants, the test refusal rate triggered its replacement with the Stair Climbing Test. The Stair Climbing Test was used for the remaining participants, and safety screening was the main reason for this test not being done. The remaining tests more frequently omitted were those requiring maximal and submaximal physical effort or hand immersion in cold water. For the tests requiring increased physical effort, we believe our standardized safety screening appropriately excluded individuals with cardiogenic symptoms or hypertension. For the tests requiring hand immersion in cold water, while excluding individuals with hypertension was supported by evidence [55, 56, 57], it is possible that our safety screening based on hand pain or changes in finger coloration was too stringent and did not necessarily indicate a circulatory or sensory condition.

This study had limitations. The physical test battery was longer than 2 h and took place at the end of the 4.5 h‐long visit. Thus, it is likely that fatigue and discomfort may have affected test performance, particularly in individuals with limited mobility for whom the assessment visit was even longer. Despite our efforts to minimize fatigue, such as thoughtfully planning the sequence of the tests, providing customized breaks, and having water and snacks available, some participants may still have experienced fatigue that impeded their performance. Moreover, our team offered flexible scheduling of visits, clearly communicated study procedures and time requirements, reimbursed participants for their time and travel, and ensured that the research staff remained grounded in ethics and empathy. Striking a balance between minimizing patient burden and extracting the data required to advance the research is challenging and requires thoughtful consideration of the potential barriers involved in participation and the unique participant's circumstances.

## 5 Conclusions

The results support the safety and feasibility of a comprehensive battery of physical tests and pain assessments in individuals with cLBP. The comprehensive characterization and reference values provided in this study can be used for future research planning and in clinical practice.

## Conflicts of Interest

The authors declare no conflicts of interest.

## Supporting information


**Data S1:** Supporting information.
