# Correction to “Feasibility of Physical Exam and Performance‐Based Tests in Individuals With Chronic Low Back Pain: A Descriptive Study”

**DOI:** 10.1002/jsp2.70205

**Published:** 2026-07-21

S. R. Piva , Z. Alfikri , W. Anderst , et al., “Feasibility of Physical Exam and Performance‐Based Tests in Individuals With Chronic Low Back Pain: A Descriptive Study,” JOR Spine 8, no. 3 (2025): e70096, 10.1002/jsp2.70096.40799632 PMC12340543

In the above article, Kevin M. Bell was incorrectly designated as the corresponding author. The correct corresponding author is Sara R. Piva (spiva@pitt.edu).

The Authorship Contributions section was omitted. The Authorship Contributions are as follows:

Conception: Sara R. Piva, Zakiy Alfikri, Charity G. Patterson. Acquisition: Jessa Darwin, Cristiane Carlesso, Marit E. Johnson, Rachel E. Roos. Analysis or Data Interpretation: Clair Smith, Gina P. McKernan, Leming Zhou, Gwendolyn A. Sowa, Nam V. Vo, William Anderst, Kevin M. Bell, Anthony Delitto, Carol M. Greco, Rachel McLoughlin, Michael J. Schneider. Reviewing, final approval, and accountability: Sara R. Piva, Zakiy Alfikri, Charity G. Patterson, Jessa Darwin, Cristiane Carlesso, Marit E. Johnson, Rachel E. Roos, Clair Smith, Gina P. McKernan, Leming Zhou, Gwendolyn A. Sowa, Nam V. Vo, William Anderst, Kevin M. Bell, Anthony Delitto, Carol M. Greco, Rachel McLoughlin, Michael J. Schneider.

We apologize for these errors.

