# Supplementary material for: Feasibility of Physical Exam and Performance‐Based Tests in Individuals With Chronic Low Back Pain: A Descriptive Study
Source: JOR Spine. 2025 Aug 12;8(3):e70096. doi: 10.1002/jsp2.70096 (PMC12340543; doi:10.1002/jsp2.70096)
Supplement: Supplementary file 1 — Data S1: Supporting information. [file JSP2-8-e70096-s001.docx]

STROBE Statement—checklist of items that should be included in reports of observational studies

|  | Item No. | Recommendation | Page  No. | Relevant text from manuscript |
| --- | --- | --- | --- | --- |
| **Title and abstract** | 1 | (*a*) Indicate the study’s design with a commonly used term in the title or the abstract | 1 | In-Field Ecological Momentary Assessment from Wearable Motion Sensors and Self-Report in a Chronic Low Back Pain Cohort |
|  |  | (*b*) Provide in the abstract an informative and balanced summary of what was done and what was found | 3 |  |
| Introduction | | | |  |
| Background/rationale | 2 | Explain the scientific background and rationale for the investigation being reported | 6 |  |
| Objectives | 3 | State specific objectives, including any prespecified hypotheses | 7 | The objective of this study was to utilize complementary self-reported EMA and wearables to characterize pain characteristics, physical activity, sedentary behavior, and sleep patterns from individuals with cLBP in the field over a seven-day period, presenting results both for the entire cohort and stratified by age (≥60 years old, <60 years old) and sex at birth (male, female). |
| Methods | | | |  |
| Study design | 4 | Present key elements of study design early in the paper | 7 |  |
| Setting | 5 | Describe the setting, locations, and relevant dates, including periods of recruitment, exposure, follow-up, and data collection |  |  |
| Participants | 6 | (*a*) *Cohort study*—Give the eligibility criteria, and the sources and methods of selection of participants. Describe methods of follow-up  *Case-control study*—Give the eligibility criteria, and the sources and methods of case ascertainment and control selection. Give the rationale for the choice of cases and controls  *Cross-sectional study*—Give the eligibility criteria, and the sources and methods of selection of participants | 8 | Participants were included if they were adults, English speakers, and had cLBP as defined by the NIH Task Force^14^—pain located between the inferior border of the ribcage and the gluteal fold for at least three months, with pain occurring on at least half the days in the past six months. Participants were excluded if they: 1) were not identified in the University of Pittsburgh Medical Center (UPMC) Electronic Health Record system, 2) were participating in a masked intervention study for LBP, or 3) had a medical condition that would place the participant at increased risk or preclude them from complying with study procedures. |
|  |  | (*b*) *Cohort study*—For matched studies, give matching criteria and number of exposed and unexposed  *Case-control study*—For matched studies, give matching criteria and the number of controls per case |  |  |
| Variables | 7 | Clearly define all outcomes, exposures, predictors, potential confounders, and effect modifiers. Give diagnostic criteria, if applicable | 8-13 |  |
| Data sources/ measurement | 8* | For each variable of interest, give sources of data and details of methods of assessment (measurement). Describe comparability of assessment methods if there is more than one group | 14 |  |
| Bias | 9 | Describe any efforts to address potential sources of bias |  |  |
| Study size | 10 | Explain how the study size was arrived at |  |  |

Continued on next page

| Quantitative variables | 11 | Explain how quantitative variables were handled in the analyses. If applicable, describe which groupings were chosen and why | 14 |  |
| --- | --- | --- | --- | --- |
| Statistical methods | 12 | (*a*) Describe all statistical methods, including those used to control for confounding | 14 | Pain intensity level, pain interference level, activity level, and sleep duration were calculated from the self-reported EMA data. Peak pain intensity profile and peak pain interference profile were also determined. From the back sensor IMU data, activity counts, wear time, activity level, and step counts were calculated. From the ActiGraph devices’ data, activity counts in the y axis, wear time, activity level, and step counts were calculated. Sleep duration was also calculated from the wrist ActiGraph. Various metrics exhibited a non-normal distribution; therefore, medians and interquartile ranges (IQR) were calculated. The medians represent the overall median across all participants, derived from their individual within-person medians over the seven-day period. Descriptive statistics for the cohort overall, and stratified by sex (male, female) and age (≥60 years old, <60 years old), are provided. |
|  |  | (*b*) Describe any methods used to examine subgroups and interactions | 14 | Descriptive statistics for the cohort overall, and stratified by sex (male, female) and age (≥60 years old, <60 years old), are provided. |
|  |  | (*c*) Explain how missing data were addressed |  |  |
|  |  | (*d*) *Cohort study*—If applicable, explain how loss to follow-up was addressed  *Case-control study*—If applicable, explain how matching of cases and controls was addressed  *Cross-sectional study*—If applicable, describe analytical methods taking account of sampling strategy |  |  |
|  |  | (*e*) Describe any sensitivity analyses |  |  |
| Results | | | | |
| Participants | 13* | (a) Report numbers of individuals at each stage of study—eg numbers potentially eligible, examined for eligibility, confirmed eligible, included in the study, completing follow-up, and analysed | 14-15 | Out of the 1,007 enrolled participants, 989 used the app to submit their EMA data. There were 398 males and 590 females. One participant reported sex at birth as intersex and was therefore not included in the male/female comparison. The age of the participants ranged from 18 to 95 years old with an average of 58.8 ± 16.5 years old, and their BMI averaged 31.5 ± 7.6 kg/m². In terms of racial composition, there were 26 (2.6%) multiracial individuals, 746 (75.4%) White individuals, 18 (1.8%) Asian individuals, 178 (18.0%) Black or African American individuals, 4 (0.4%) individuals classified as Other, and 17 (1.7%) individuals of unknown or undisclosed race. At enrollment, the participants reported a mean pain intensity of 5.4 (SD 2.1) and median pain intensity of 5 (IQR 3) on a 0-10 numeric pain rating scale, and a mean PROMIS Pain Interference T-score of 60.5 (SD 7.5) and median PROMIS Pain Interference T-score of 61.2 (IQR 9.6). |
|  |  | (b) Give reasons for non-participation at each stage |  |  |
|  |  | (c) Consider use of a flow diagram |  |  |
| Descriptive data | 14* | (a) Give characteristics of study participants (eg demographic, clinical, social) and information on exposures and potential confounders | 14-23 |  |
|  |  | (b) Indicate number of participants with missing data for each variable of interest |  |  |
|  |  | (c) *Cohort study*—Summarise follow-up time (eg, average and total amount) |  |  |
| Outcome data | 15* | *Cohort study*—Report numbers of outcome events or summary measures over time |  |  |
|  |  | *Case-control study—*Report numbers in each exposure category, or summary measures of exposure |  |  |
|  |  | *Cross-sectional study—*Report numbers of outcome events or summary measures |  |  |
| Main results | 16 | (*a*) Give unadjusted estimates and, if applicable, confounder-adjusted estimates and their precision (eg, 95% confidence interval). Make clear which confounders were adjusted for and why they were included | 14-23 | Figures 4-9, Tables 2 and 3 |
|  |  | (*b*) Report category boundaries when continuous variables were categorized |  |  |
|  |  | (*c*) If relevant, consider translating estimates of relative risk into absolute risk for a meaningful time period |  |  |

Continued on next page

| Other analyses | 17 | Report other analyses done—eg analyses of subgroups and interactions, and sensitivity analyses |  |  |
| --- | --- | --- | --- | --- |
| Discussion | | | | |
| Key results | 18 | Summarise key results with reference to study objectives | 23-27 |  |
| Limitations | 19 | Discuss limitations of the study, taking into account sources of potential bias or imprecision. Discuss both direction and magnitude of any potential bias | 23-24 |  |
| Interpretation | 20 | Give a cautious overall interpretation of results considering objectives, limitations, multiplicity of analyses, results from similar studies, and other relevant evidence | 24 |  |
| Generalisability | 21 | Discuss the generalisability (external validity) of the study results |  |  |
| Other information | |  | | |
| Funding | 22 | Give the source of funding and the role of the funders for the present study and, if applicable, for the original study on which the present article is based | 30 | The Back Pain Consortium Research Program is administered by the National Institute of Arthritis and Musculoskeletal and Skin Diseases. This research was supported by the National Institutes of Health through the NIH HEAL Initiative under award number U19AR076725-01. The content is solely the responsibility of the authors and does not necessarily represent the official views of the National Institutes of Health or its NIH HEAL Initiative. |

*Give information separately for cases and controls in case-control studies and, if applicable, for exposed and unexposed groups in cohort and cross-sectional studies.

**Note:** An Explanation and Elaboration article discusses each checklist item and gives methodological background and published examples of transparent reporting. The STROBE checklist is best used in conjunction with this article (freely available on the Web sites of PLoS Medicine at http://www.plosmedicine.org/, Annals of Internal Medicine at http://www.annals.org/, and Epidemiology at http://www.epidem.com/). Information on the STROBE Initiative is available at www.strobe-statement.org.
